# Fast Retinal Vessel Detection and Measurement Using Wavelets and Edge Location Refinement

**DOI:** 10.1371/journal.pone.0032435

**Published:** 2012-03-12

**Authors:** Peter Bankhead, C. Norman Scholfield, J. Graham McGeown, Tim M. Curtis

**Affiliations:** Centre for Vision and Vascular Science, Queen's University Belfast, Belfast, Northern Ireland; National Microelectronics Center, Spain

## Abstract

The relationship between changes in retinal vessel morphology and the onset and progression of diseases such as diabetes, hypertension and retinopathy of prematurity (ROP) has been the subject of several large scale clinical studies. However, the difficulty of quantifying changes in retinal vessels in a sufficiently fast, accurate and repeatable manner has restricted the application of the insights gleaned from these studies to clinical practice. This paper presents a novel algorithm for the efficient detection and measurement of retinal vessels, which is general enough that it can be applied to both low and high resolution fundus photographs and fluorescein angiograms upon the adjustment of only a few intuitive parameters. Firstly, we describe the simple vessel segmentation strategy, formulated in the language of wavelets, that is used for fast vessel detection. When validated using a publicly available database of retinal images, this segmentation achieves a true positive rate of 70.27%, false positive rate of 2.83%, and accuracy score of 0.9371. Vessel edges are then more precisely localised using image profiles computed perpendicularly across a spline fit of each detected vessel centreline, so that both local and global changes in vessel diameter can be readily quantified. Using a second image database, we show that the diameters output by our algorithm display good agreement with the manual measurements made by three independent observers. We conclude that the improved speed and generality offered by our algorithm are achieved without sacrificing accuracy. The algorithm is implemented in MATLAB along with a graphical user interface, and we have made the source code freely available.

## Introduction

Variations in blood vessel diameters occur as part of the autonomous control of blood flow in healthy subjects and at different stages in the pulse cycle [1], while sustained changes may also indicate the presence of some pathologies [2]. Measurements of vessel calibre are therefore of interest both to physiologists looking to better understand the regulation of blood flow [3], [4] and to clinicians interested in the prediction, diagnosis or progression of disease [5]–[7]. Of particular importance are retinal images, as these may be used to directly visualise blood vessels non-invasively in vivo [2], [6]. However, accurate quantification of changes in vessel calibre is difficult to automate fully because of large variations in image type, size and quality. In practice, measurements are frequently obtained using semi-automated computer-assisted methods [8]–[10], which can be both laborious and open to user-bias.

### Retinal vessel segmentation

Fully automating the analysis of vessel calibre in still images relies firstly upon accurately locating the blood vessels. The application of state-of-the-art image processing techniques to the accurate segmentation of vessels in human fluorescein angiograms and fundus (red-free) images has received considerable attention in recent years [2], [11]. Published retinal segmentation algorithms can be broadly categorised as those that require training images and those that do not. The former group comprises primarily supervised algorithms that use a set of hand segmented images (in which pixels are manually identified either as belonging to vessels or not) to train a classifier to distinguish vessel pixels according to feature vectors computed, for example, from neighbouring pixel intensities or colour channel information [12], wavelet coefficients [13] or filter correlations [14], [15]. When training images are not used, algorithms typically work by preprocessing the image to enhance the contrast between vessels and the background, before a binarisation step (e.g. thresholding) is applied. Preprocessing may be achieved using matched filtering, which involves filtering the image with a family of 1D filters derived from Gaussian functions (chosen to model the profile across most vessels) rotated at different angles, then retaining the largest magnitude response [16]–[18]. In order to better enhance vessels of different widths, the width of the filter may be varied in addition to its orientation, resulting in a multiscale matched filter method [18], [19]. Other filter-based approaches include using a Laplace kernel [20], and multiple applications of 2D Gaussian smoothing at different scales followed by ridge detection [12], [21]. The effectiveness of morphological, rather than linear, filters for vessel detection has also been explored [22].

In general, algorithms that integrate the use of training images and classifiers report better segmentation results at the cost of higher computation times. The requirement for training images can be considered a drawback, because manual segmentation of even a single image is a difficult and time-consuming process, open to inter-user variability [12] – although this can be somewhat mitigated if hand-segmenting only a portion of an image is sufficient to train the classifier [23]. The main strength of these supervised algorithms is that, because of the complexity of retinal images combined with the high level of variability arising from acquisition conditions and the health of the subject, in many cases it may not be possible to transform or enhance an image in such a way that a simple thresholding operation can reliably identify the vasculature – and so the more sophisticated decision making processes used by classifiers may help to overcome this. On the other hand, unsupervised algorithms are often faster, and can be tested easily on new image types without any need for training sets to be generated. Their primary disadvantage is that they often use filters and operations that are tailored for a particular type or resolution of image and can require significant modifications to be applied to others; for example, matched filters of a fixed size are unlikely to perform well on both low and high resolution images. In some cases, the fundamental approach of preprocessing followed by thresholding typically used with unsupervised algorithms has been combined with the automatic optimisation of parameters (such as filter sizes and thresholds), but this then reintroduces the requirement to have manually segmented training images [24], [25].

### Vessel diameter measurement

One might suppose that all of the important information regarding the retinal vasculature is encoded in the binary images produced by an accurate vessel segmentation algorithm. If these binary images could be fully deciphered, a completely automated analysis of the retinal vasculature would be possible. However, despite the proliferation of vessel segmentation algorithms, relatively little attention has been given to converting this information directly into vessel diameter measurements [26]. Two notable exceptions are ‘Vessel Finder’ [27] and ‘Retinal Image multiScale Analysis’ (RISA) [28], both of which have been applied to the analysis of retinal images of infants at risk of retinopathy of prematurity (ROP). However, both of these pieces of software offer only mean diameters for each vessel segment, rather than individual measurements along the vessel length. Also, by making measurements directly from the segmented images, the final results may be unduly influenced by thresholds used in the binarisation stages [27].

In large-scale studies, it is common to use a computer-assisted method to measure vessels [29], [30]. This requires a user to manually draw a line perpendicularly across a vessel, before edge points are located from the vessel profile using, for example, thresholds [31] or gradients [9]. Interpolation may be used to improve the precision of measurements over those computed directly from segmented pixels, although the user may introduce bias by his or her choice of measurement locations and angles, and the time taken to draw each profile line restricts the number of diameters obtainable.

A more sophisticated approach to measurement is to track the vessel boundaries. From initial seed points along vessel centrelines – which may be identified manually [2] or automatically [32] – a pair of vessel edge points and an orientation can be identified. Based upon these, a pixel intensity profile is computed slightly further along the vessel, and the edge points detected from this profile are used to update the orientation [33]. Various tests can be applied whenever the tracking breaks down, so that a vessel that fades from view for several pixels may still be correctly identified. Alternatively, if one uses active contours for tracking, a contour is initialised (manually or automatically) close to an edge before an iterative algorithm applies forces to the contour in an effort to draw it towards the edge. While the definition of appropriate forces to apply to the contour may be difficult, and the algorithm used to apply these forces is somewhat slow and so inappropriate for the interactive processing of large image sets, this method offers the important advantage of being able to continue to track vessels even if they fade briefly from view within the image, and it is possible to evaluate diameters as the distance between the edges at any location. Active contours are used for fully-automated vessel segmentation by the *Extraction of Segment Profiles* (ESP) algorithm [26].

Finally, an alternative, graph-based algorithm has recently been described [34]. To begin, a ‘vesselness map’ generated by filtering the image is thresholded to give a binary image [14], which is cleaned up and thinned to provide vessel centrelines. Vessel orientations are identified by principal component analysis of several adjacent centreline pixels. Profiles computed across these centrelines are then used to build a graph, which is searched to determine vessel edges by minimising a cost function. A smoothness constraint ensures that the edges are feasible.

### Contribution of the current work

In this paper, we describe a fast and accurate unsupervised algorithm to detect and measure blood vessels in retinal images. This involves two main steps. The first is simple approach for vessel segmentation by thresholding wavelet coefficients, which we introduce here and demonstrate to be much faster than other unsupervised segmentation methods, while achieving comparable accuracy. The second step consists of a new alternative to the graph-based algorithm to extract centrelines and localise vessel edges from image profiles, by making use of spline fitting to determine vessel orientations and then searching for the zero-crossings of the second derivative perpendicular to the vessel. Using the fundus photographs contained within a standard image database and extracting diameter measurements from the detected edges, we show that our entire algorithm is capable of achieving a high level of accuracy and low measurement error, with a much shorter processing time than that required by the other state-of-the-art vessel analysis algorithms (ESP and graph-based), both for low and high resolution images.

Finally, we have made the MATLAB implementation of our algorithm available online, along with a graphical user interface, manual, source code and all test functions. This software is suitable for a range of image types without a need for prior training, including both fundus photographs and fluorescein angiograms, and can be further customised with the addition of new algorithms.

## Methods

### Image sources

We obtained human retinal images from publicly available databases. The source of fundus images used to test the segmentation was the DRIVE (Digital Retinal Image for Vessel Extraction) database [12]. The forty colour images are 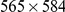 pixels in size, and were captured in digital form using a Canon CR5 nonmydriatic 3CCD at 45 field of view as part of a screening programme in the Netherlands. Two sets of manually segmented binary images showing blood vessels were made available by these authors. To test calibre measurements, we used the recently published REVIEW (REtinal Vessel Image set for Estimation of Widths) database [35]. These images are of higher resolution than the DRIVE images, ranging in size from 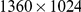 to 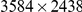 pixels. In all cases, colour images were converted to grayscale by extracting the green channel information and treating this as containing gray levels, because the green channel exhibits the best contrast for vessel detection [17].

### Vessel segmentation by wavelet thresholding

**Figure 1:**
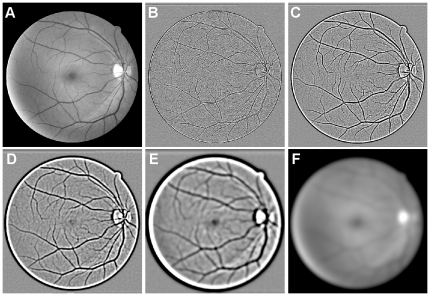
Wavelet levels and the final scaling coefficients calculated from four iterations of the IUWT. (A) The original image from the DRIVE database. (B–E) Wavelet levels 1–4, computed using the IUWT algorithm. Wavelet coefficients have been scaled linearly for display, so that light and dark pixels indicate positive and negative coefficients respectively, while zero is represented by a mid-tone gray. (F) The smooth residual image. Adding this residual to all the wavelet levels would reconstruct the original image.

The Isotropic Undecimated Wavelet Transform (IUWT) is a powerful, redundant wavelet transform that has been used in astronomy [36] and biology [37] applications. It affords a particularly simple implementation that can be readily appreciated without recourse to wavelet theory: at each iteration 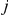, scaling coefficients 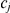 are computed by lowpass filtering, and wavelet coefficients 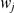 by subtraction [38]. The scaling coefficients preserve the mean of the original signal, whereas wavelet coefficients have a zero mean and encode information corresponding to different spatial scales present within the signal. Applied to a signal 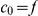, subsequent scaling coefficients are calculated by convolution with a filter 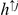.
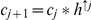
where 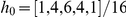 is derived from the cubic B-spline, and 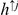 is the upsampled filter obtained by inserting 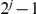 zeros between each pair of adjacent coefficients of 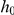. If the original signal 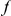 is multidimensional, the filtering can be applied separably along all dimensions. Wavelet coefficients are then simply the difference between two adjacent sets of scaling coefficients, i.e.
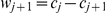
Reconstruction of the original signal from all wavelet coefficients and the final set of scaling coefficients is straightforward, and requires only addition. After the computation of 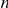 wavelet levels,
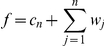



The effect of applying the IUWT to a fundus image from the DRIVE database is shown in Fig. 1. The set of wavelet coefficients generated at each iteration is referred to as a wavelet level, and one may see that larger features (including vessels) are visible with improved contrast on higher wavelet levels. Segmentation can then be carried out very simply by adding the wavelet levels exhibiting the best contrast for vessels and thresholding based upon a percentage of the highest (if applied to an angiogram) or lowest (if applied to a fundus image) valued coefficients. The thresholds should be computed from pixels within the field of view (FOV) only, in order to ensure that the dark pixels outside this do not contribute to the threshold chosen; if a FOV mask is not available, one can normally be produced by simply applying a global threshold to the image. This is best applied to the red channel of a colour fundus photograph.

**Figure 2:**
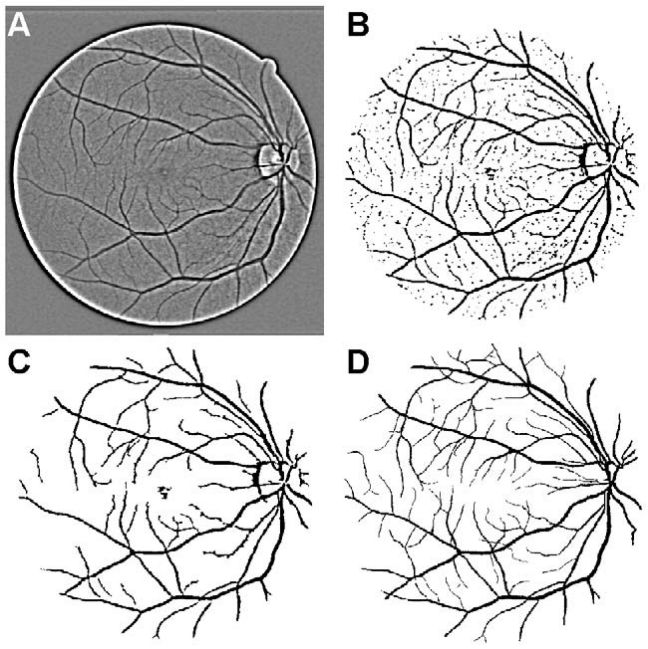
Thresholding wavelet coefficients of the IUWT. (A) The sum of wavelet levels 2 and 3. (B) A threshold was applied to A to identify the lowest 15% of wavelet coefficients within the FOV. (C) A cleaner version of the segmentation in B, created by removing connected objects and filling holes with areas smaller than 75 and 20 pixels respectively. (D) A hand-segmented image from the DRIVE database, shown for reference.

The choice of wavelet levels and thresholds do not typically need to be changed for similar images; indeed, in all cases for fundus images (both low and high resolution) we set the threshold to identify the lowest 20% of coefficients as vessels, and varied only the choice of wavelet levels if the image sizes were different. Because the percentage of vessel pixels within the FOV is more typically around 12–14% (as determined using manually segmented images), the thresholded image is likely to be oversegmented (i.e. many non-vessel pixels have been misclassified as vessels). However, the majority of the vasculature is represented by one large connected structure in the binary image, whereas misclassified pixels tend to be clustered to form isolated objects. These small objects can be removed simply based upon their area, either in terms of pixels or a proportion of the image size. Similarly, small holes present within thresholded regions can be filled in. Most remaining erroneous detections are removed during later processing steps. The results of this segmentation applied to a fundus photograph from the DRIVE database are shown in Fig. 2.

### Centreline computation

The next step is to apply a morphological thinning algorithm [39]. Thinning iteratively removes exterior pixels from the detected vessels, finally resulting in a new binary image containing connected lines of ‘on’ pixels running along the vessel centres. The number of ‘on’ neighbours for each of these pixels is counted: end pixels (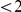 neighbours) are identified, and branch pixels (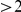 neighbours) are removed. The removal of branches divides the the vascular tree into individual vessel segments in preparation for later analysis. This is useful because diameters are not well-defined at branches, and also because diameters measured before a significant branch or bifurcation are not directly comparable with those measured afterwards, as less blood will flow through the vessel afterwards and there will be a drop in pressure.

The elimination of as many uninteresting centrelines as possible at this stage helps to improve the speed of the later processing steps. To this end, centrelines are first cleaned up by removing short segments (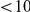 pixels). Because any of these short segments that contained end pixels were likely to be spurs, which often occur as an unwanted side-effect of thinning, their corresponding branch pixels are replaced to avoid causing the main vessels to which they were connected being erroneously subdivided. A coarse estimate of vessel diameters is then calculated using the distance transform of the inverted binary segmented image. This gives the Euclidean distance of every ‘vessel’ pixel from the closest non-vessel pixel, and therefore doubling the maximum value of the distance transform along the thinned centrelines provides an estimate of the diameter of every vessel segment at its widest point. A centreline is removed if it contains fewer pixels than its estimated diameter, since such centrelines are unlikely to correspond to measureable vessels.

Each remaining connected group of pixels now corresponds to the centreline of a potential vessel segment that is suitable for further analysis.

### Centreline refinement using spline fitting

The orientation of a vessel segment at any point could be estimated directly from its centreline, but discrete pixel coordinates are not well suited for the computation of angles. A least-squares cubic spline (in piecewise polynomial form) is therefore fitted to each centreline to combine some smoothing with the ability to evaluate accurate derivatives (and hence vessel orientations) at any location. A parametric spline curve is required, with appropriate parameterisation essential to obtain a smooth centreline. For this we used the centripetal scheme described by Lee [40].

Adjusting the spacing of the breaks between polynomial pieces in the spline can give some control over a preference for smoothness or the ability to follow complex shapes, although we found a spacing of approximately 10 pixels between breaks performed acceptably on all tested images. The precise break spacing can vary because the vessel segment is divided into polynomial pieces of equal length, and the segment length is unlikely to be an exact multiple of the polynomial piece length. If the number of data points is very low, a single cubic polynomial is fit to the centreline instead.

### Image profile generation

Image (pixel intensity) profiles using linear interpolation are then determined from the raw, grayscale image perpendicularly to the spline at any point along the vessel, with approximately one pixel intervals being selected as a suitably high resolution. An image-dependent problem arises when determining the length of the profiles, which need to be longer in images containing wider vessels. To overcome this we again use the diameters estimated from the distance transform above. By creating image profiles that are at least double the largest diameter estimate we can be confident that the profiles will be long enough to stretch beyond even the widest vessels, and also allow additional space for later filtering.

The image profiles are finally aligned side by side to create ‘straightened’ vessel images in which each row is a separate profile. Two corresponding sets of binary profiles are also generated by appling the same method to the segmented image and using nearest neighbour interpolation. The first set contains only ‘vessel’ pixels that form a connected region that overlaps the centreline, and this set therefore defines an initial estimate of the location of the vessel within the image profiles. The second set of binary profiles contains pixels outside the FOV along with any other vessel pixels that do not overlap the centreline (and so correspond to neighbouring vessels), and is used later to define regions within the profiles where vessel edges should not be found.

### Vessel edge identification

**Figure 3:**
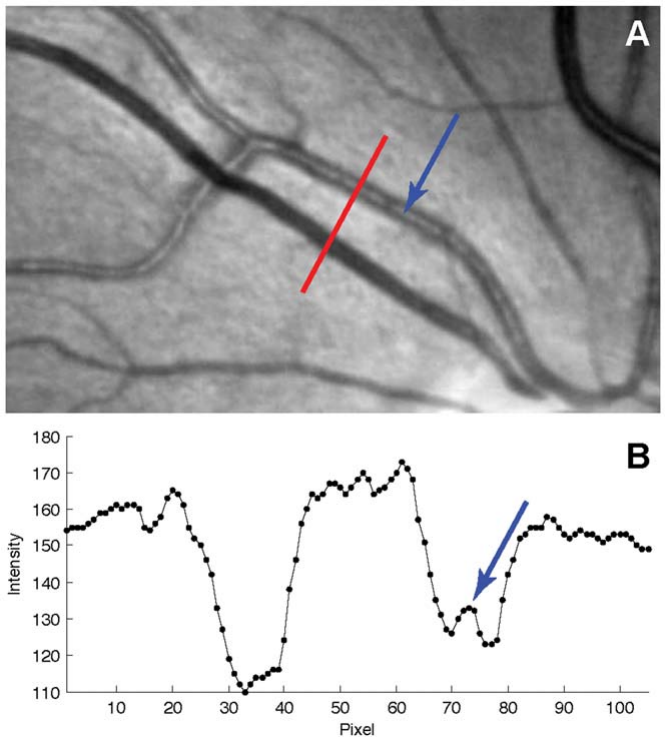
The central light reflex. (A) Part of an image from the REVIEW database containing a pronounced central light reflex, which is the bright region seen running through one of the vessels (blue arrow). (B) The pixel intensity profile computed along the red line shown in A. Here, the central light reflex appears as a small ‘hill’ in the rightmost vessel.

The measurement of diameters requires the location of edge points, but these have no single ‘natural’ definition within the image space. Vessel profiles in fundus and fluorescein angiography images resemble Gaussian functions, and edges have previously been defined in a variety of ways, including using gradients or model fitting [41]. One of the main complications encountered when trying to develop a general vessel diameter measurement strategy is the possible presence of the ‘central light reflex’ [42], which is seen as a ‘dip’ or ‘hill’ approximately in the centre of the vessel profile, and which is more likely to be found in higher resolution images and wider vessels (Fig. 3). Its origins are unclear, although it is thought to emanate from the column of densely packed erythrocytes moving through the retinal microvasculature [43]. The marked enhancement of the light reflex may be of clinical interest; for example, it appears to be associated with hypertension, although further investigation and a more objective quantification of changes are needed [43]. That some vessel measurement algorithms have misidentified the light reflex as the vessel edge has been reported as problematic [41], [44], and explicit strategies for dealing with this issue are required to ensure that any measurement is sufficiently robust [41], [45]–[47].

Here, we define an edge as occurring at a local gradient maximum (the rising edge) or minimum (the falling edge), as identified to sub-pixel accuracy using the zero-crossings of the second derivative. We have adopted a four-step method to identify these edges for each vessel:

1. Estimate the average vessel width from the binary profiles. The sum of ‘vessel’ pixels in each profile is computed, and the median of these sums is taken as the provisional width.
2. Compute an average of all the vessel profiles (omitting pixels previously identified as belonging to other vessels or outside the FOV), and identify the locations of the maximum and minimum gradient to the left and right of the centre respectively, bounded to a search region of one estimated diameter from the centre. These locations give the columns in the vessel profile images at which edges are predicted to fall. The distance between the two columns also gives a more refined and robust estimate of mean vessel width, largely independent of the thresholds used for the initial segmentation.
3. Apply an anisotropic Gaussian filter to the vessel profiles image to reduce noise, and then calculate a discrete estimate of the second derivative perpendicular to the vessel by finite differences.
4. Identify locations where the sign of the pixels in each filtered profile changes, and categorise these based upon the direction of the sign change into potential left and right vessel edges. Using connected components labelling, link the possible edges into distinct trails. Remove trails that never come within 1/3 of an estimated vessel diameter from the corresponding predicted edge columns. The final edges are then the zero-crossings belonging to the longest remaining trails to each side of the vessel centre, and the diameter is simply the Euclidean distance between these edges.

**Figure 4:**
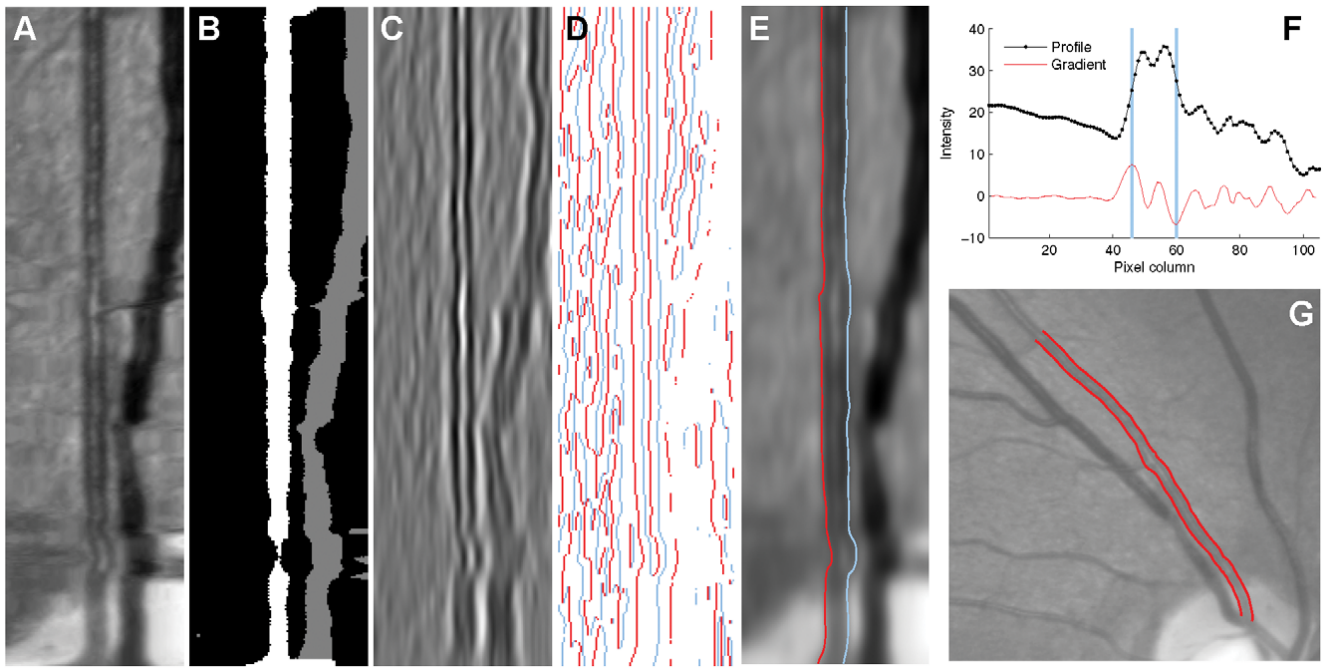
Determining vessel edges by zero-crossings. (A) A ‘straightened’ vessel image, created by stacking many image profiles alongside one another. (B) Corresponding stacked profiles determined from the initially-segmented image. White pixels belong to the vessel under consideration, while gray pixels belong to other detected vessels. An initial vessel width estimate is determined from the median of the sum of white pixels on each row, and refined using the averaged profile in F. (C) The profiles in A after smoothing with an anisotropic Gaussian filter, and subsequently applying a second filter to approximate the second derivative computed perpendicular to the vessel (i.e. horizontally). In this representation most of the vessel consists of negative values, but the central light reflex contains positive values. (D) Pixels in C representing positive-to-negative (red) and negative-to-positive (blue) transitions. Transitions corresponding to the second vessel region in B are removed. The length of each connected line is computed, and only the longest lines that fall close to the estimated vessel boundaries are retained. (E) The edges identified by the algorithm, superimposed on top of the straighted vessel. (F) A mean vessel profile, computed by averaging all the profiles in A, excluding pixels belonging to other vessels. The locations of the maximum and minimum gradients to the left and right of centre are shown in blue. The transitions in D are removed if they do not fall close to these locations, and the distance between them is also used when calculating the Gaussian filter sizes. (G) The edges identified by the algorithm, shown on the original image from the REVIEW database.

This process is summarised in Fig. 4. In the ideal case, a single trail of suitable zero-crossings will exist to the left and right of the vessel centre and edge identification is straightforward. The additional tests are intended to produce reasonable results whenever the edge may be broken, while avoiding misclassifying zero-crossings due to the central light reflex or other image features. The smoothing in the third step deals with the sensitivity to noise of computing approximations of derivatives applied to discrete data. The horizontal and vertical sigma values 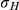 and 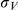 of the Gaussian filter are calculated by scaling the square root of the estimated widths 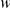 produced by the previous step, and therefore more smoothing is applied to vessels with larger diameters. The scaling parameters may be adjusted according to image noise, but we used 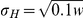 and 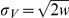 for all images. Because this smoothing is applied to the stacked image profiles rather than the original image, the filter is effectively oriented parallel to the vessel at each point. This ensures that most blurring occurs within or alongside the vessel – rather than in all directions, which might have otherwise affected edges or merged vessels with neighbouring structures.

### Algorithm summary

**Figure 5:**
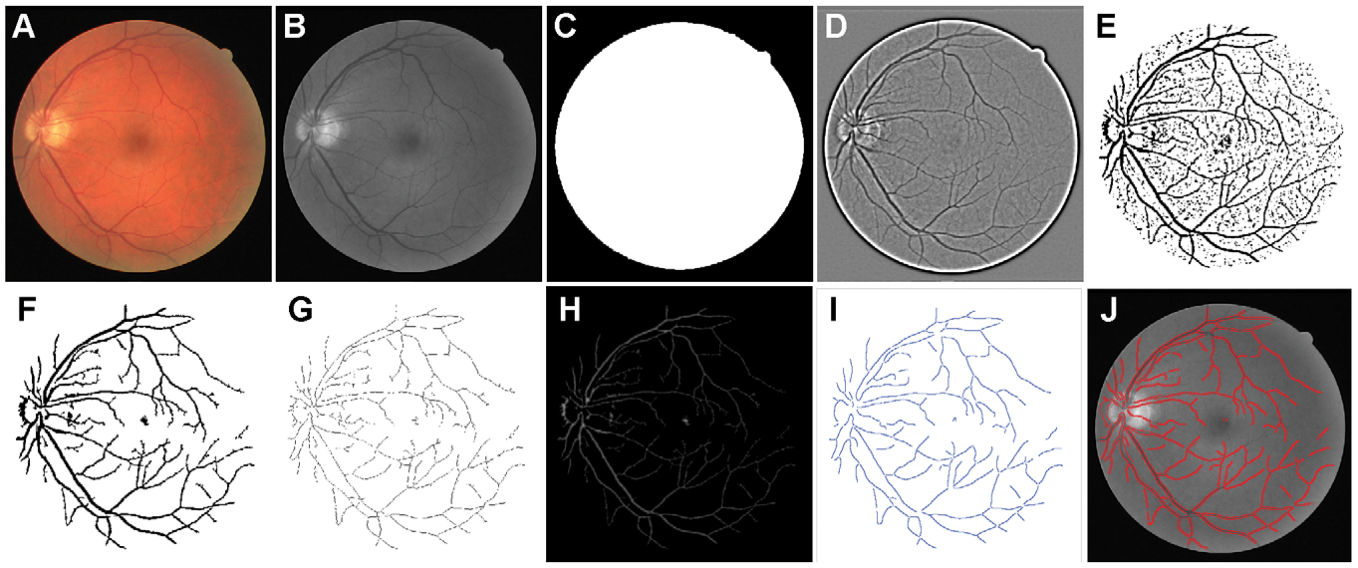
Overview of the main steps taken by our algorithm when processing a fundus image. (A) The image (here, from the DRIVE database) is read. (B) The green channel is selected for later processing. (C) A mask is produced by thresholding. (D) The IUWT is applied to B. (E) Wavelet coefficients are thresholded. (F) Small objects are removed and holes are filled in E. (G) Morphological thinning is applied to F. (H) The distance transform is applied to F to assist with estimating diameters and removing erroneously detected segments. (I) Branches are removed from G and spline fitting applied to determine centrelines. (J) Edges are detected perpendicular to the centrelines.

The main steps of the algorithm are illustrated in Fig. 5.

## Results

### Segmentation accuracy

The IUWT is somewhat atypical as a wavelet transform, and has a particularly straightforward implementation. It effectively provides an efficient means of combining background subtraction along with noise and high-frequency content suppression using an approximately Gaussian filter – so that the wavelet coefficients resemble the values that would be computed directly using a ‘difference of Gaussians’ filter. Nevertheless, despite its simplicity we found it to be well-suited to the task of vessel detection. Although accurate segmentation is only a means to an end in the algorithm described here, and does not constitute the final output, in order to establish the suitability of the IUWT for efficient vessel detection we have compared it with more specialised published algorithms.

The true positive rate (TPR) refers to the proportion of vessel pixels identified by a segmentation algorithm that coincide with vessel pixels in ‘ground truth’ segmented images, while the false positive rate (FPR) is the proportion of detected pixels not considered vessels in the ground truth images. Relatedly, the ‘accuracy’ is a single value frequently quoted for comparison, defined as the number of correctly assigned pixels in the segmented image (either vessel or non-vessel) divided by the total number of pixels within the FOV. In common with other papers, we used the ‘test’ set of images from the DRIVE database for evaluation, treating the first set of manually segmented images as the ground truth and reporting the average TPR, FPR and accuracy results of all 20 images. Although masks depicting the FOV are included with the DRIVE database, for our segmentation implementation we used a FOV mask computed simply by thresholding the raw DRIVE images with a fixed threshold value of 20 before applying a morphological erosion using a 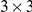 square structuring element. This provided more accurate FOVs than those offered in the DRIVE database, and we used our FOVs to compute the percentage threshold values and to define the region of interest in which vessels could be detected by our algorithm. Nevertheless, we used the DRIVE database FOVs when computing TPR, FPR and accuracy scores in order to ensure comparability with previous results.

Table 1 shows the results for our IUWT segmentation along with those reported for previous unsupervised retinal segmentation algorithms tested using the DRIVE database. Supervised algorithms tend to perform better, but at the cost of greater computation time and the requirement to have hand-segmented images available. Here, we applied our IUWT algorithm with the following settings: the sum of wavelet levels 2 and 3 was thresholded to identify the lowest 15% of coefficients, before objects smaller than 75 pixels were removed and holes smaller than 20 pixels were filled. Note that the values reported in the table for our algorithm were not the ‘best’ accuracy scores possible: performing an iterative search using the hand-segmented images for the parameters that would optimise the accuracy scores was found to produce a very minor improvement, but this was not sufficient to change the ranking of the IUWT segmentation in the table. Therefore we report rounder parameter values that are more likely to be found in practice when training images are not present.

**Table 1:** Vessel segmentation algorithm accuracy.

Method	TPR	FPR	Acc.
Second observer	0.7760	0.0275	0.9473
Ricci [15]	–	–	0.9563
Mendonca [22]	0.7315	0.0219	0.9463
**IUWT 15%, 75/20 px**	**0.7027**	**0.0283**	**0.9371**
Garg [56]	–	–	0.9361
Espona [57]	0.7436	–	0.9352
Martinez-Perez [21]	0.7246	0.0345	0.9344
All background	0	0	0.8727

Comparison of the accuracy of unsupervised vessel segmentation algorithms as applied to the DRIVE image database. The hand segmented images from the first manual observer are used as the benchmark. True and false positive rates (TPR and FPR) are included where these were made available in the original papers. Note that assigning all pixels to the background – i.e. detecting no vessels at all – still achieves an accuracy score of 0.8727. On the other hand, a second manual observer achieved an accuracy of 0.9473. The accuracy of segmentation algorithms can therefore be expected to fall within this range; improving on the accuracy score of the second observer is not necessarily beneficial, since the choice of the first observer as the benchmark is arbitrary.

It can be seen from Table 1 that this simple IUWT approach performs comparably to more complex specialised vessel segmentation algorithms. The lower TPR of the IUWT as compared to other algorithms is the result of fewer very narrow vessels being located – although this is somewhat compensated for by a low FPR, so that the overall accuracy is not compromised. It is worth noting that, because of the effects of blur and noise, the sufficiently accurate quantification of changes in the diameter of vessels that are only 1–2 pixels wide cannot be expected. If one wishes to measure such small vessels, higher-resolution images would be required.

#### Efficiency of segmentation

We have implemented the algorithm using MATLAB (R2011a, The MathWorks, Natick, MA), using only the functions offered by MATLAB and its Image Processing Toolbox (i.e. no additional compiled ‘mex’ code was used to optimise the speed of computationally expensive parts of the algorithm). MATLAB is widely used when creating new retinal image analysis algorithms because of its range of built-in functions that facilitate algorithm development, although in some cases code performance can be improved by using a lower-level language such as C++ [48]. Nevertheless, we found vectorised MATLAB code to perform well in terms of processing time.

The proper comparison of algorithm efficiency is difficult because, in general, source code has not been made publicly available to allow testing on the same machine using the same conditions (e.g. operating system, or MATLAB version where appropriate). One must then resort to using the information included in the published papers that have made use of the same images. Table 2 provides a summary of the reported processing times for algorithms tested using the DRIVE database images. Because timing and test system information is often omitted from papers, only two other algorithms that do not require training images are included.

**Table 2:** Vessel segmentation algorithm times.

Method	Processor	RAM	Implementation	Training	Accuracy	Time
**IUWT**	**2.13 GHz**	**2 GB**	**MATLAB**	***No***	**0.9371**	**0.093 s**
Al-Rawi [24]	1.7 GHz	–	MATLAB	Yes	0.9420	2.156 s
Anzalone [25]	2.40 GHz	192 MB	MATLAB	Yes	0.9419	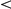 6 s
Espona [57]	1.83 GHz	2 GB	C++	*No*	0.9352	38.4 s
Mendonca [22]	3.2 GHz	960 MB	MATLAB	*No*	0.9463	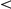 150 s
Soares [13]	2.1 GHz	1 GB	MATLAB	Yes	0.9466	180 s
Staal [12]	1 GHz	1 GB	MATLAB	Yes	0.9441	900 s

Comparison of segmentation times for vessel segmentation algorithms applied to a DRIVE database image. Timings are reported in the original papers, and details of computer specifications and implementation languages are given where these were made available. Four of the algorithms required training images to achieve their accuracy scores. Timings are given only for processing individual images; one-off initialisation stages required by some algorithms (e.g. to train a classifier) are not included. The IUWT algorithm made use of wavelet levels 2 and 3.

From these results, it is clear that the IUWT segmentation is considerably faster, and the discrepancy in speed is unlikely to be explained by differences in test systems. Indeed, recently some attention has been given to implementing vessel segmentation using specialist hardware. An algorithm implemented for the SCAMP-3 vision system achieves an accuracy of score of 0.9180 [49], while an alternative algorithm making use of Cellular Neural Networks reports an accuracy of 0.9261 [50]. However, the accuracy of the IUWT strategy is higher than both of these, and the IUWT is more suitable for scaling to images of different resolutions.

### Validation of diameter measurement accuracy

#### Comparison with manually segmented images

**Figure 6:**
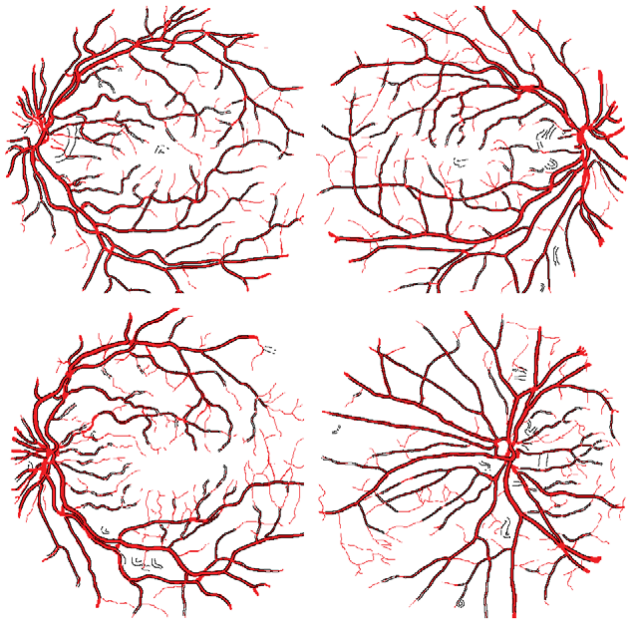
Application of vessel detection to DRIVE database images. Overlays of detected edge points (black) applied to the first four images in the DRIVE database test image set, superimposed on the corresponding manually segmented images (red).

The similarity between vessel diameters in ‘ground truth’ manually segmented DRIVE database images and the diameters measured entirely by our algorithm cannot readily be quantified. The segmented image produced by the IUWT method will differ from the manual segmentation, which will cause measurement locations and angles not to match up. One may, however, observe good agreement for wider vessels by overlaying the vessel edge points located by our software on top of the manually segmented images (Fig. 6). As noted above, very narrow vessels (1–2 pixels in diameter) are consistently ignored by our algorithm, and higher resolution images would be required to measure these.

#### ‘Measurement error’ and the REVIEW database

In order to evaluate the reliability of vessel diameter measurements, we made use of the images included in the REVIEW database [26], [35]. This comprises 3 Image Sets containing full fundus images: high-resolution (HRIS), vascular disease (VDIS) and central light reflex (CLRIS), with each set containing representative images that are particularly large, show visible pathologies and have vessels exhibiting prominent central light reflexes respectively. A fourth set, the kick-point image set (KPIS), contains downsampled high-resolution images of several large-diameter non-tortuous vessels. The database also offers manual diameter measurements made by 3 independent observers using a custom software tool for marking vessel edge points, so that the ground truth diameters are considered to be the average of the measurements made by the 3 observers at the same location in a vessel segment. A total of 5066 locations are available. For comparison of results, the error is then defined as
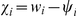
where 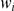 is a single width measured by the algorithm being tested, and 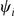 is the ground truth measurement at the same location in the image. Performance is evaluated by considering the standard deviation of the error, denoted 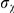. The justification for this is that there is no single ‘correct’ vessel edge definition, and one is primarily interested in changes in diameter determined using the same method; with this in mind, a consistent measurement bias can be corrected but fluctuations in the error cannot [35].

The authors of the database have used it to validate their Extraction of Segment Profiles (ESP) algorithm [26]. Additionally, they have implemented four previously used methods of edge point location:

1. *Gregson:* a rectangle is fitted to a vessel intensity profile, and the width is set so that the area under the rectangle is equal to the area under the profile [51].
2. *Half Height Full Width (HHFW):* the standard half-height method, which uses thresholds set half-way between the maximum and minimum intensities to either side of an estimated centre point [45].
3. *1D Gaussian (1DG):* a 1D Gaussian model is fit to the vessel intensity profile [52].
4. *2D Gaussian (2DG):* similar to the 1D Gaussian case, but the model is extruded into 2D [47].

In Table 3 we have reproduced their results as reported in [26] and supplemented these with the results obtained by applying the same tests to the output of our algorithm and those published for the graph-based method [34]. For reproducibility, the relevant parameters used by our algorithm were: *Wavelet levels:* 3–4 (VDIS & CLRIS), 2 (KPIS & HRIS downsampled), 3–5 (HRIS original); *Threshold:* 20%; *Minimum object size:* 0.05%; *Fill hole size:* 0.05%; *Centreline spur & short segment removal length:* 10 pixels; *Spline piece spacing:* 10 pixels; *Parallel smoothing scale factor:* 2, *Perpendicular smoothing scale factor:* 0.1.

**Table 3:** REVIEW database comparison.

	KPIS			CLRIS			VDIS			HRIS		
Method	%	Mean	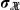	%	Mean	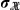	%	Mean	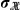	%	Mean	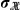
Standard	100	7.52	0.00	100	13.80	0.00	100	8.85	0.00	100	4.35	0.00
O1	100	7.00	0.23	100	13.19	0.57	100	8.50	0.54	100	4.12	0.29
O2	100	7.60	0.21	100	13.68	0.70	100	8.91	0.62	100	4.35	0.26
O3	100	7.97	0.23	100	14.52	0.57	100	9.15	0.67	100	4.58	0.28
Gregson	100	7.29	0.60	100	12.80	2.84	100	10.07	1.49	100	7.64	1.48
HHFW	96.3	6.47	0.39	0	–	–	78.4	7.94	0.88	88.3	4.97	0.93
1DG	100	4.95	0.40	98.6	6.30	4.14	99.9	5.78	2.11	99.6	3.81	0.90
2DG	100	5.87	0.34	26.7	7.00	6.02	77.2	6.59	1.33	98.9	4.18	0.70
ESP	100	6.56	0.33	93.0	15.7	1.47	99.6	8.80	0.77	99.7	4.63	0.42
Graph	99.4	6.38	0.67	94.1	14.05	1.78	96.0	8.35	1.43	100	4.56	0.57
**Our algorithm**	**100**	**6.30**	**0.29**	**100**	**14.27**	**0.95**	**99.0**	**8.07**	**0.95**	**99.5**	**4.66**	**0.32**

REVIEW database comparison of successful measurement percentages (i.e. the percentage of vessel locations at which a meaningful measure of vessel diameter was returned by the algorithm), mean vessel diameters and standard deviations of the measurement error (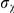). The data included in the top part of the table are reproduced from [26] (© 2009 IEEE), to which we have added the results of the graph-based algorithm [34] and those obtained by applying the same tests using our algorithm. O1–O3 were obtained from measurements made by three manual observers, and ‘Standard’ is the average of these measurements.

When interpreting the results in Table 3, two additional points should be made:

- The HRIS images were downsampled by a factor of 4 before being input into the test algorithms, and it is these downsampled measurements that are reported in the REVIEW database [26]. Because manual measurements were made on the original images, vessel widths are considered to be known to an accuracy of 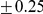 pixels (discounting human error). Although the lower computational requirements of our algorithm make it feasible to measure full-resolution images within an acceptable time frame, we report the results using similarly downsampled images for comparability with the results given elsewhere.
- The earlier edge location algorithms are initialised with centre point locations and angles as determined from the ‘ground truth’ measurements; a measurement success percentage less than 100% indicates that the algorithm did not produce a meaningful result (e.g. it did not converge). In contrast, the ESP, graph-based and our algorithm incorporate vessel detection along with measurement, and so are provided with the original images only. Consequently, a reduction in the measurement success percentage in these cases may indicate that the vessel was not detected. When determining comparable measurements for our algorithm, we first associated each ground truth centre point with the closest detected centre point. We kept the association only if the distance between both points was less than or equal to the true vessel diameter at that location, and also if the detected point was not closer to another ground truth point. These strict criteria ensured that each detected point was counted only once. However, in some cases the ground-truth points had a spacing less than one pixel and so not all could be uniquely matched with detected points even when the detection was successful. These caused a slight decrease in our reported success percentages, particularly in the HRIS.

**Figure 7:**
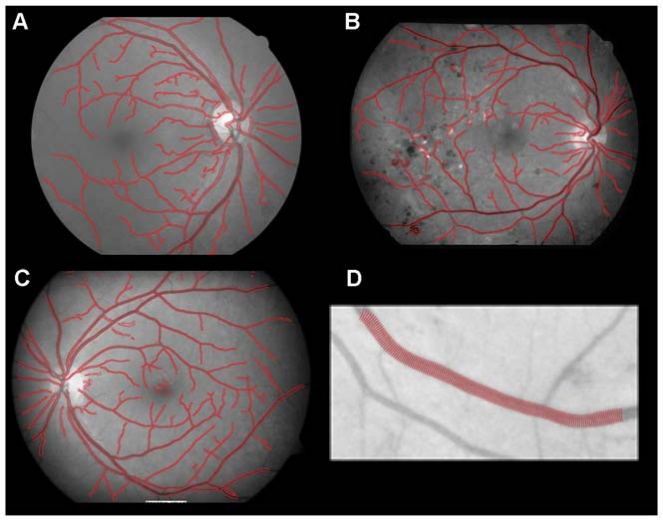
Application of vessel detection to REVIEW database images. (A–C) Vessels detected in example images from the CLRIS, HRIS and VDIS respectively. (D) Individual diameters found for a vessel in a KPIS image. Note that in the KPIS image, the visible branching vessels are much narrower and dimmer in comparison to the main vessel, so that they occur as unconnected objects in the segmented image. This difference in contrast then allows edges still to be found for the main vessel at these branching locations.

All three of the most recent methods – graph-based, ESP and our algorithm – outperform the other edge location algorithms both in terms of reducing 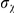 and providing mean diameter estimates more consistently close to the ‘ground truth’. The most distinct improvement offered by our algorithm was seen in the CLRIS, and probably arises because progressively refining edge estimates helped to ensure that the discontinuities caused by the central light reflex were rarely confused with the true vessel edge. Performance on the VDIS is comparatively weaker. This is a considerably noisier dataset than the others, and increasing the smoothing scale parameters would improve the results by reducing the noise accordingly. However, increased smoothing has a slight negative effect upon the measurements made in cleaner images, and we chose to report the results keeping as many parameters as possible the same across images of different resolutions and quality to demonstrate that careful parameter tuning on a by-image basis is not necessary. In this regard, it is important to note that even in this worst case the 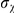 remains under 1 pixel. Example processed images from each image set are shown in Figure 7.

#### Processing times

The biggest difference between our approach and other algorithms tested using the REVIEW database is in the length of time required to process an image. Computation times for the MATLAB implementation of our algorithm tested on two different systems are given in Table 4. A DRIVE database image requires approximately 1 second to process, while a 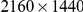 pixel image image takes around 3–7 seconds depending upon the computer specifications. These results compare favourably with timings reported for the other algorithms: 11 minutes to process a DRIVE image using the ESP method (1.2 GHz Pentium, 1GB RAM) [26], and several minutes for a 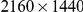 pixel image using the graph-based algorithm (composed of the vessel detection step, plus 50 seconds for the graph creation and solving; system information not given) [34].

**Table 4:** Total image analysis times.

Image source	Size	Time for system 1	Time for system 2
DRIVE	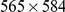	1.12 s	0.65 s
REVIEW: VDIS	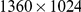	4.72 s	2.10 s
REVIEW: CLRIS	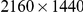	7.14 s	3.00 s
REVIEW: HRIS (downsampled)	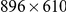	2.12 s	0.98 s
REVIEW: HRIS	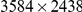	25.07 s	9.32 s

Mean computation times for our entire vessel analysis algorithm applied to a range of images using two different test systems. System 1: 2.13 GHz Intel Core 2 Duo PC with 2 GB RAM, running Windows XP Professional and MATLAB R2010a 32-bit. System 2: 3.07 GHz Intel Xeon Workstation with 16 GB RAM, running Windows 7 Professional and MATLAB R2011a 64-bit.

## Discussion

Until recently, the study of retinal vessel diameters for clinical purposes has remained largely a research tool because it is laborious, although improvements in computerised analysis have the potential to change this [5]. For truly automated analysis to be feasible, the software used must be robust regarding variations in image quality and the presence in an image of other signs of pathology. The algorithm described here is general enough to offer a practical alternative to manual measurements for a wide range of studies, while offering important benefits in terms of speed and repeatability.

### Use of the IUWT for segmentation

Previously, more sophisticated multiscale algorithms for retinal image segmentation, such as the supervised method of Soares et al. [13], have reported good results, but have been criticised for requiring long computation times [25]. Although the wavelet transform is used for multiscale analysis, one might contend that the IUWT segmentation described above is not a true multiscale detection algorithm because detection does not occur separately on each wavelet level; rather, levels are first combined by summation before thresholding. While presented in the language of wavelets, this segmentation is therefore equivalent to applying limited smoothing for noise reduction, before subtracting a much more highly smoothed version of the image that approximates the inhomogeneous background present in retinal images, and then thresholding the result. The smoothing filters used are approximately Gaussian in shape. However, formulating the segmentation in terms of the IUWT enables a intuitive method whereby the algorithm may be adapted to images of differing resolutions.

The justification for this approach rests upon the goals of efficiency, generality and user-friendliness. Smoothing using the IUWT may be computed much more quickly than using a large Gaussian filter because of the many zero coefficients used in the filters. Furthermore, the IUWT provides a convenient framework for varying the levels of smoothing. The filter sizes are built in to the definition of the IUWT; while using other filters with sizes finely tuned to a particular image may provide some improvements in segmentation accuracy, it is much faster and more intuitive for a user to choose from, say, 6 possible wavelet levels rather than any arbitrary Gaussian filter size. Finally, adding the wavelet levels before segmenting means that the user is able to compare the effects of different thresholds easily by looking at the wavelet level sum and thresholded binary image. This makes it possible to quickly and interactively test the appropriateness of our software for analysing new images.

### Choice of algorithm parameters

Although a relatively large number of parameters are associated with our algorithm, in practice we found that many of these can be left at default values. For example, because wavelet coefficient and object removal thresholds are defined using the FOV size, and smoothing filter sizes are automatically adapted to vessel widths, the same values for the related parameters can be used across a wide range of images. In fact, the only parameter we needed to adjust for any of our measurement tests, in which the images ranged from 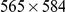 to 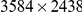 pixels, was the choice of wavelet levels. This directly relates to the size of structures that should be detected, making it possible to give a preference for detecting narrow or wide vessels, and therefore the most appropriate choice depends upon the image dimensions and capture angle (i.e. the extent of the retina contained within the image). However, once chosen, the same wavelet levels were used for all images with similar resolutions.

### Algorithm efficiency

The efficiency, and not only accuracy, of segmentation algorithms is of great importance if the software is to be of practical use. The test images most commonly used are from the DRIVE [12] and STARE [17] databases, which contain images that are 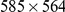 and 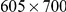 pixels in size respectively. However, these are very small compared to the high-resolution images often used in practice for clinical purposes, which may be 15 times larger or more. An analysis requiring minutes for a single DRIVE database image may be acceptable, but greatly increased computational requirements of large images might mean that downsampling is the only feasible option, at a cost of spatial information. This is not necessary with our approach, with which full-resolution images in the HRIS required average processing times of 9.19 and 25.19 seconds for our faster and slower test system respectively. Omitting downsampling also slightly improved the analysis accuracy for the HRIS, giving a 99.96% success rate, mean diameter of 4.38 pixels and 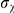 of 0.29.

In determining the overall analysis time the efficiency of the segmentation step is an important factor. The speed of the IUWT segmentation is of course reduced whenever larger images are processed: more wavelet levels (and, consequently, filtering operations) are required, and larger contiguous areas of memory need to be found to store the images. However, because of the many zero coefficients in the filters, the effects are less dramatic than they would be using alternative techniques or more dense filters. Furthermore, by using the segmented image only to extract centrelines and thereby shifting the refinement of accurate vessel edge location to the measurement stage, we were able to reduce computation time without sacrificing overall reliability. Consequently, the time required for the entire analysis remained lower than that of alternative algorithms implementing segmentation alone (Tables 2 and 4).

### Locating vessel edges from zero-crossings of the second derivative

Although a comparative study of Gaussian fitting, Sobel operators and sliding linear regression filters deemed the last to provide the most consistent edge localisation from image profiles, the use of linear regression filters required a minimum vessel width of at least 10 pixels [41]. This criterion was frequently not met in our test images, and so restricts the general usefulness of the approach. Perhaps the most common recent strategy when determining a vessel edge has been to fit a model [47], [52]–[55]. When the light reflex is present, a single Gaussian function is no longer an adequate model for the vessel profile, and so double [41], [53] or piecewise Gaussian [54] models have been proposed. Generally, this consists of two Gaussian functions – one to represent the vessel and another to model the light reflex. However we did not adopt this approach because of three main problems:

1. Depending upon the complexity of the model, computation times can be greatly increased.
2. For high-resolution fluorescein angiograms in particular, if pixels are saturated then the true profile can be flattened at the top, which can affect the fit.
3. The fit may depend upon the influence of pixels that extend beyond the vessel itself, and is therefore affected by the length of the profile line. A longer image profile permits more pixels from outside the vessels to influence the fit. The background itself is inhomogeneous, and can differ on each side of the vessel, although models typically assume it is approximately constant and flat.

The approach we have described does not suffer from these problems and can be computed quickly. Making measurements from the binary image directly would be insufficient to obtain reliable measurements, since these would be heavily dependent upon the precise threshold used (and potentially other image features). However, by using a coarse estimate of the vessel width based upon the initial segmentation we are able to search for a high local gradient magnitude only in the region surrounding the likely vessel edge. The estimate also makes it possible to smooth the image in a scale-dependent manner without requiring the application of an additional multiscale transform at the measurement stage, although a more thorough exploration of the estimated vessel widths and the optimal smoothing parameters could improve this further. While speed and efficiency were primary considerations when choosing to adopt this strategy, the low 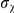 in Table 3 suggests that these do not come at a cost of accuracy or repeatability when compared to more complex edge computations.

Nevertheless, one important limitation of our method is that if the image contrast decreases then appropriate zero-crossings may not be found at all locations along the vessel. Interpolation could be used in such cases, although currently we prefer not to report any results where the algorithm could not identify suitable crossings. Integrating aspects of another, more complex algorithm to deal with the most difficult measurements into our approach may lead to better overall performance, while retaining fast processing for identifying the main vessels. For example, an active contour initialised from the edges provisionally given by our algorithm would automatically combine interpolation with a smoothness constraint that prevents identifying the wrong zero-crossings, and so overcome regions of reduced contrast. Because the initialisation should already be close to the edges, the contour should converge relatively quickly.

### Algorithm availability

The MATLAB implementation of our algorithm is included as supporting information (File S1) along with a user manual and description of how to run the tests reported in this paper. These files can also be downloaded from http://sourceforge.net/p/aria-vessels. For sample data, the DRIVE and REVIEW databases are available at http://www.isi.uu.nl/Research/Databases/DRIVE/ and http://reviewdb.lincoln.ac.uk/ respectively.

### Conclusion

The algorithm described here fully automates the analysis of retinal vessel diameters. It allows the fast calculation of diameters all along the length of each vessel rather than at specific points of interest, thereby producing more fine-grained results than would be possible manually or using interactive, computer-assisted software. Computation time per image is typically no more than several seconds on a current PC, and large images can also be processed without a need for downsampling.

## Supporting Information

File S1 **MATLAB implementation of the vessel analysis algorithm described in this paper, along with documentation.**
(ZIP)
