# Supplementary material for: Fast Retinal Vessel Detection and Measurement Using Wavelets and Edge Location Refinement
Source: PLoS One. 2012 Mar 12;7(3):e32435. doi: 10.1371/journal.pone.0032435 (PMC3299657; doi:10.1371/journal.pone.0032435)
Supplement: File S1 — MATLAB implementation of the vessel analysis algorithm described in this paper, along with documentation. (ZIP) [file pone.0032435.s001.zip › ARIA/ARIA user guide.pdf]

ARIA: Automated Retinal Image Analyzer v1.0

Pete Bankhead

Developed at:
Centre for Vision and Vascular Science, Queen's University of Belfast, UK

Current address:
Nikon Imaging Center @ Heidelberg University, Germany

9th December 2011

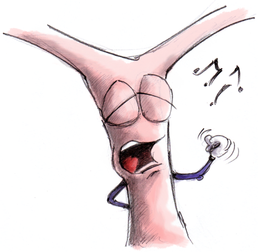

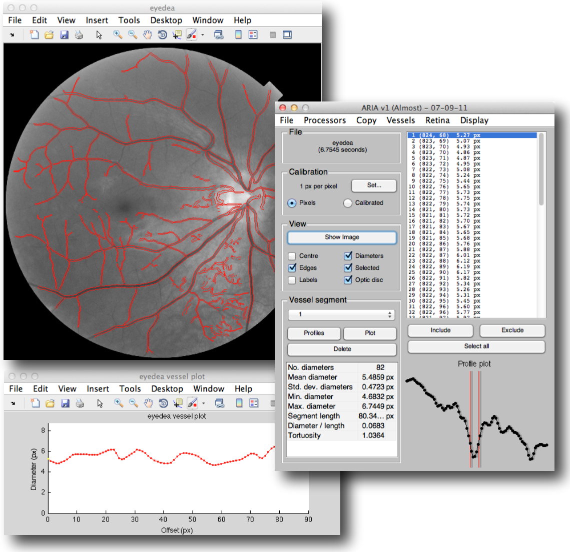

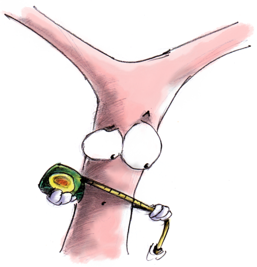

Contents

1 Introduction **2**

2 Getting started **2**
2.1 Running ARIA through MATLAB 2
2.2 The main idea 3
2.3 Analyzing an image 3
2.4 The user interface 4

3 Tips and tests **5**
3.1 Important settings 5
3.2 Improving the results 7
3.3 Test data 8

4 Notes for developers **9**
4.1 Understanding the source code 9
4.2 Designing new vessel processors 11

1 Introduction

Automated Retinal Image Analyzer (ARIA) is software designed for the automated detection and measurement of retinal blood vessels. It is based upon an algorithm that has been tested using both fundus photographs and fluorescein angiograms, and which might also be usefully applied to other non-retinal 2D images in which tubular structures should be measured.

The software is made available along with its source code, and can easily be extended by modifying the code or adding new algorithms. One algorithm for detection and analysis is built in. The main focus when developing this has been to devise a method that is fast and general enough that it can be applied to a wide range of images, while also requiring relatively few settings to be changed for the effective processing of different images. It should also be possible either to determine sensible setting from knowledge of the images, or to interactively explore different options to find those most appropriate.

While ARIA is freely available, note that it can only be run from within MATLAB™. It therefore requires a valid MATLAB installation and license (version R2010a or later – earlier versions have not been tested), including its associated Image Processing Toolbox.

2 Getting started

2.1 Running ARIA through MATLAB

After first starting up MATLAB, use the top bar or `Current Folder` pane to navigate to the base folder containing the ARIA files (or add this folder to the path under `File` → `Set Path...`). Then type `ARIA` in the command window, or right-click the file `ARIA.m` and choose `Run`. After a brief delay the ARIA user interface should appear. To avoid needing to manually navigate to the correct folder each time, type `ARIA_setup` and the locations will be saved.

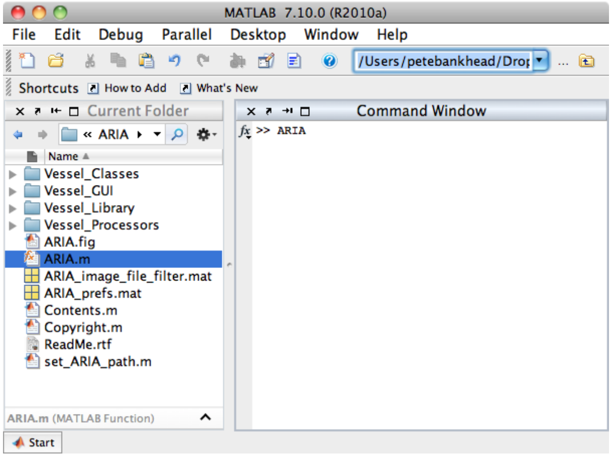

Figure 1: Running ARIA from the MATLAB command prompt.

2.2 The main idea

Two things are needed to analyze an image automatically:

1. A MATLAB function that implements the analysis algorithm
2. A number of arguments (also called parameters or settings) required by the function

In ARIA, both the name of the function and the required arguments are saved as *processors*. This makes it possible to have different processors for different image types, e.g. low or high resolution, fundus photographs or fluorescein angiograms. Two processors might use exactly the same function for analysis, but differ in their function arguments. In general, the same processor should be used for similar images if their measurements will be compared later.

2.3 Analyzing an image

The main processing in ARIA happens immediately when an image is opened, and so before choosing an image file it is important to check that the desired processor has been selected in the `Processors` menu of the user interface.

Selecting the `Processors` → `Prompt for settings` option means that the software will prompt for any required arguments (e.g. vessel detection thresholds, whether the vessels are lighter or darker than the background) so that the analysis can be tailored for the current image. Otherwise, the most recent arguments associated with the processor are used and the analysis happens automatically[1]. After identifying suitable arguments using several images, it is best to turn off the prompts for all similar images to ensure that the analysis is unbiased and repeatable.

Available processors are stored in the `Vessel_Processors` folder. You can make a new one from within ARIA with `Processors` → `Create new processor`, but it may be easier just to go into the folder, duplicate an existing processor and rename it. It should then appear in the menu the next time ARIA is run. It can be modified by opening an image with the `Prompt for settings` option selected.

[1]Note that this assumes the author of the processor has supported this option – it is quite possible to write a processor that ignores this value, and perhaps always prompts or never prompts. Also, it might not be possible to automate all steps – for example, if a file is needed to use as a field of view mask, then the appropriate file name will need to be chosen each time.

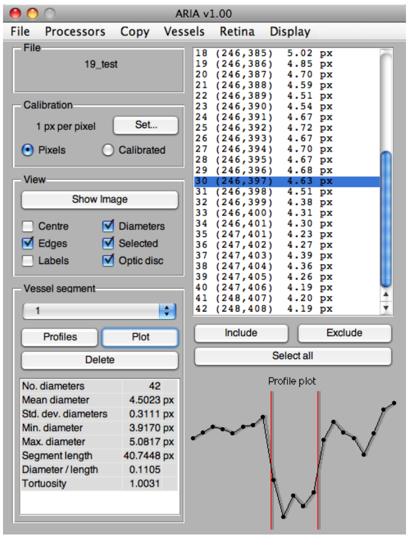

Figure 2: The main window of the ARIA user interface.

2.4 The user interface

Most options and features of ARIA can be accessed through the main window that appears when it is run, either from the menu or the buttons and check boxes, without requiring any MATLAB programming skills. The following subsections describe the various options visible in the user interface.

2.4.1 The menus

File Includes commands to open a new image or save the results of analyzing the currently-opened image so that these may be reopened in a subsequent ARIA session. If you are familiar with MATLAB, you might want to use `Send to workspace` in order to gain access to the underlying data structures. Then you can do much more processing using MATLAB's commands and toolboxes.

Processors Whenever an image is opened, the selected processor determines what happens next. The processor defines which function should be used for processing, and what arguments should be passed to it.

Copy Copy either the individual diameter measurements corresponding to the currently-selected vessel segment, or the summary measurement table (number of diameters, mean, standard deviation, etc.).

Vessels Sort the vessel segment labels according to segment length or mean diameter. This makes it easier to identify and remove segments with extreme measurements, where these are more likely to result from false detections or incorrect measurements.

Retina Manually mark the location of the optic disc, and display circles to show regions surrounding the optic disc. This is useful for some protocols, in which only measurements

within fixed distances from the optic disc are required.

Display Adjust the colours and other properties connected to display. If the user interface is sluggish when displaying the image, turning off double buffering may help (at the expense of some flickering).

2.4.2 The main window

Calibration Change the calibration value (pixel size) of the image. By default, measurements are given in pixels. If the calibration is set, the related pixel measurements are scaled by this value, and the required calibration unit is shown alongside measurements.

View Show the image, and toggle which lines are displayed. See also the `Display` menu for further customization. Turning on the `Labels` option shows the vessel segment numbers, which are useful for selecting a specific vessel segment in the next box.

Vessel segment Look in more detail at one individual vessel segment, or delete the entire segment if it is not required. `Plot` gives a plot of diameters against distance along the segment centre line (see also the `Display` → `Plot` options). `Profiles` displays the individual vessel profiles computed along the vessel (at approximately 1 pixel intervals) stacked together. This can also be understood as an image giving a 'straightened' view of the vessel. Various summary measurements are also given. These are computed from the vessel diameters that are designated as 'included' (see next section). (`Segment length` differs slightly: it is the sum of the Euclidean distance between all points along the centre line, from the first to the last 'included' diameters, and incorporating all centre points in between, irrespective of the inclusion status.) The `Tortuosity` measure is the `Segment length` divided by the Euclidean distance between the vessel segment end points. It is therefore equal to 1 for a perfectly straight vessel segment, and higher for a more tortuous vessel.

Vessel diameters The right-hand side of the main user interface window gives a list of the diameters for the currently-selected vessel segment. If a diameter is selected in this list, the corresponding profile plot is displayed at the bottom, along with marked vessel edge locations. If multiple diameters are selected, the average of all profiles (without edges) is shown instead. You can also select one or more diameters and set whether these are 'included' or 'excluded'. Excluded diameters are not used for the summary measurements, and are displayed on the image and in plots in a different colour. They are also omitted whenever `Copy` → `Vessel diameters` is clicked. This makes it possible to manually remove spurious measurements.

3 Tips and tests

3.1 Important settings

When running the built-in analysis algorithm in `Prompt for settings` mode, there are a lot of potential settings to change. Most can be left to their default values, and the results are likely to be similar even if they are varied a bit, but some are more sensitive or useful. In order of importance, these are:

- `Dark vessels` – During the segmentation phase you need to specify whether the vessels are darker than the background (fundus images) or lighter (fluorescein angiograms). This settings affects whether vessels are detected at all, although determining its correct value should be easy.

- `Wavelet levels` – These should be set according to image resolution and the size of vessels that should be detected; larger vessels require levels with higher numbers. The preview images demonstrate how the output will look for different choices. This will determine which vessels are detected, and whether 2 thin parallel tracks are considered to be parallel vessels or the same vessel with a central light reflex.

- `Threshold percentage` – This defines the percentage of pixels on the wavelet levels that will be provisionally detected as vessels. Often, in typical human retinal images about 12-14% of the pixels belong to vessels, but the default value for this settings is 20% – which means about one third of the detected pixels will not actually belong to vessels. However, many of these extra pixels will belong to small, isolated objects that are not very vessel-like, and which will be removed by later steps of the algorithm. Therefore it is a good idea to set this threshold value to be slightly higher than the percentage of pixels you would ideally detect in any image.

- `Apply connectivity constraint` – Edges are detected where there is a steep gradient around the vessel. Most of the time the steepest gradients near to a vessel correspond to its true edges, but sometimes they might belong to other vessels or structures. Several constraints are already integrated into the detection algorithm, but these might not be sufficient to ensure the correct edges are found. The 'connectivity constraint' forces the algorithm to only accept large gradients that form a long, connected line close to the vessel – and thus it ignores most other spurious gradients, which are normally much shorter. However, sometimes this constraint is too much, and even the true edge does not meet it (perhaps due to noise, pathology or imperfections in the detected centre line). Therefore it is a good idea to set this to `Yes` if doing so gives reasonable results, but `No` if it causes too many vessels to be missed. Because edges are *defined* in the same way irrespective of this setting (i.e. as zero-crossings of the second derivative) then in ideal conditions the results are identical whether or not the constraint is used.

- `Perpendicular smoothing scale` – This changes the amount of smoothing to apply across the vessel before searching for edges. If this is too large, neighbouring vessels may become merged and the edges poorly localized; however, if it it too small then noise may cause problems for edge localization and the detected vessel edge will be broken or missing in parts. Technically, if the scaling is s and an estimate for the vessel width w, then a 1D Gaussian filter with $\sigma = \sqrt{sw}$ is applied to the vessel. Therefore if the vessel is 10 pixels in diameter and $s = 0.2$, it follows that $sigma \approx 1.414$. As a guide, consider values of s around 0.1–0.2, and only increase if the results are inadequate.

- `Parallel smoothing scale` – Similar to the perpendicular smoothing scale, but the 1D Gaussian filtering is applied parallel to the vessel. It can therefore take a larger value, because it is not likely to cause blurring into neighbouring vessels. However, very large values will reduce variability along edges – and so rapid changes in vessel width would be smoothed away (although such changes often indicate noise rather than actual variability

in the vessel, in which case smoothing gives a result closer to the truth). As a general guide, consider values in the range 0.5–2.

- `Clear branches` – The main effect of selecting `Yes` for this is that centre lines will be shortened approaching branches. So if you really need measurements there, you should choose `No`. But often these measurements are not so important, and are also less accurate (because the branch causes the vessel edge to 'disappear' on one side, and can pull the centre line in the wrong direction briefly). Also, the analysis is usually faster if `Yes` is selected, since less effort is spent on making more difficult measurements.

3.2 Improving the results

This section gives some common specific problems and how they might be solved by adjusting settings.

Individual vessels have two parallel centre lines

This is probably due to the presence of the 'central light reflex' (CLR) making the vessel appear as two. Try changing the wavelet levels used for the initial segmentation to use higher levels, or fewer low levels, e.g. if levels 2–4 are used, try 2–5 or 3–4. This causes more smoothing before detection, which tends to blur out the CLR so it stops causing problems. The disadvantage is that it will also blur out very small true vessels, so these become harder to detect.

Detected vessel edges deviate far from the true edge, or disappear

This might be due to noise in the image, or reduced local contrast. Try changing the setting for `Apply connectivity constraint`, or increasing the values of the `Smoothing scale` parameters.

The centre lines of vessels are subdivided, despite there being no obvious branches

The segmentation is probably inaccurate. Either you can try to adjust this by changing wavelet levels or thresholds, or alternatively increase the value of `Length of spurs to remove`. This latter option gets rid of smaller offshoots from vessels that arise from segmentation errors, but are not actually true branches. (Note that if the centre line remains, but edges disappear, then the problem is edge and not vessel detection – in which case adjusting the smoothing scales is more likely to be helpful.)

The centre line moves outside tortuous vessels

The spacing between spline breaks might be too much. Try reducing the `Spacing between spline pieces in pixels` option (although this should remain > 5). This causes more polynomial curves to be fit along the length of the vessel, making it easier to trace complex shapes. But it increases the risk of the next problem...

The angles across which the vessel diameters are measured flucuate rapidly

The spacing between spline breaks might be too little. Try reducing the `Spacing between spline pieces in pixels` option. With fewer polynomial pieces tracing the centre line, it

cannot bend as quickly and so is less prone to excessive variation caused by noise or segmentation errors.

The processing time is too long, or there is not enough memory

Try downsampling (shrinking) the image prior to processing. If the region of the image in which you are interested is small, you could crop it first (e.g. using ImageJ) making sure to leave a large enough border since vessels close to the image boundary cannot be measured. Some improvements can also be attained by reducing the number of vessels analyzed, e.g. by increasing the values for `Delete all objects` < (during segmentation) and `Minimum number of pixels for a centre line`. Finally, newer releases of MATLAB may well include more highly-optimized versions of some functions used by ARIA, so updating to the most recent MATLAB release can improve performance.

There are too many short vessels, so it's hard to see the interesting ones

Increase the value of `Minimum number of pixels for a centre line` during detection, or choose `Vessels` → `Delete` → `Few diameters` afterwards. If some short vessels may be of interest, choose `Vessels` → `Sort by...` → `Length` to move the shorter vessels to the end of the list, making them less obtrusive.

There appears to be missing information in the centre of the field of view

If you are using a mask created by thresholding, check the settings used for this. Pixels below a low threshold and above a high threshold are excluded. If the high threshold is anything less than the maximum pixel value in the image, then the bright parts of the image will be masked out. This might be helpful if you want to exclude the optic disc, for example, but most of the time it is undesirable.

No vessels are detected – just the regions close to them

If you have a fundus image, make sure that the `Dark vessels` is checked in the segmentation stage. If you have a fluorescein angiogram, make sure it isn't.

3.3 Test data

We have tested ARIA using images from three publicly available databases of fundus photographs.

High-resolution images, including manual vessel diameter measurements:

- The REVIEW database: `http://reviewdb.lincoln.ac.uk/`

Lower-resolution images, primarily used to validate vessel segmentation:

- The DRIVE database: `http://www.isi.uu.nl/Research/Databases/DRIVE/`
- The STARE database: `http://www.parl.clemson.edu/stare/probing/`

Information about how to replicate the results given in the published paper is provided in `README.txt`.

4 Notes for developers

The idea underlying ARIA's use of 'vessel processors' is that analyzing an image requires a function that can take an image and determine the location of any vessels, along with (usually) a number of arguments required by the function. The analysis can be changed by modifying the function, the arguments, or both.

By storing the function name and arguments all within a single processor file, it is easy to test out new approaches by creating new processors, and without needing to modify any existing ARIA code. These may simply be duplicates of existing processors with only a few changes. Nevertheless, to do this properly it is helpful to know a bit about how the current code is structured.

4.1 Understanding the source code

4.1.1 ARIA and object oriented MATLAB

ARIA makes extensive use of the objected oriented programming (OOP) features introduced in MATLAB 2008a. Briefly, OOP assists in the writing of short, structured and maintainable code. One of the principal concepts is 'encapsulation', which relies upon

- *Classes*, which define properties and functions for a particular purpose
- *Objects*, which are specific instances of classes

To illustrate the utility of OOP, suppose one wishes to store the following information about each vessel in a particular image:

- Coordinates of edge points (the sides of the vessel)
- Diameter measurements

In addition, one wishes also to be able to plot the diameters. There would be several ways to achieve this in MATLAB, but the 'simplest' methods might well involve storing the diameters in an array and writing a separate function for appropriate plotting. Whenever multiple vessels are stored, and more properties and functions are required, this can rapidly become complicated. OOP helps out by making it possible to define a `Vessel` class that includes all the relevant properties and functions. From this class definition, which acts as a kind of template, one can create as many vessel objects as needed (an 'object is an instance of a class'). Properties are accessed straightforwardly in a form such as `vessel.side_1`, `vessel.side_2` and `vessel.diameters`. The diameters do not actually need to be stored as an array, but are rather just calculated as needed as the distance between the two sides – and so if the sides change, one does not need to worry about updating the diameters too. Functions are also defined for the class, and available to any objects made from that class. These are called in a similar way to how properties are accessed, e.g. `vessel.plot`, or alternatively using the more familiar MATLAB syntax `plot(vessel)`. The function need only be written once, and both syntaxes automatically work.

In short, OOP in MATLAB requires a bit of a learning curve, but in the end can result in simpler code. A reader unfamiliar with this approach can find an introduction online at `http://www.mathworks.com/products/matlab/object_oriented_programming.html`.

4.1.2 Directory structure

The following subdirectories of the main ARIA directory contain code related to different aspects of the software.

Vessel_GUI

Files related to the particular user interface of the ARIA software, e.g. to change display colours.

Vessel_Classes

Four classes that relate to different aspects of vessel analysis.

- `Vessel_Data` The class that contains everything related to a single image. This includes the original image itself, any segmented (binary) image created from it, and an array containing any available vessel segments. When an image is opened, it is added to a new `Vessel_Data` object, and the processing function adds all the other extra properties required by the object.
- `Vessel_Data_IO` A simple class containing static functions to save and load `Vessel_Data` objects. It can also be made responsible for creating the `Vessel_Data` and calling the processing function whenever an image is first read.
- `Vessel` The class that defines an individual vessel segment. This contains the edge points for the vessel, as well as the coordinates of the pixels along its centre line. It also stores the profiles across the vessel, and computes diameters.
- `Vessel_Settings` A class used to store the display settings for ARIA. These are saved in the file `ARIA_prefs.mat` after the software is closed.

Vessel_Processors

The directory containing vessel processors. On startup, the names of all processors within this directory are added to the `Processors` menu of ARIA, with underscores replaced by spaces. The directory also contains `load_vessel_processor.m` and `save_vessel_processor.m`, the functions to load and save the processors in the correct format and location.

Vessel_Algorithms

A directory to store `.m` files that take care of all the steps of vessel detection, and which can therefore be associated with vessel processors. When making a new processing through the ARIA user interface, this directory is checked for available functions.

Vessel_Library

A collection of `.m` files containing functions that carry out a particular stage of processing, e.g. image segmentation, centre line fitting or diameter edge point location. These tend to take a `Vessel_Data` object as input, and set various properties during execution.

Vessel_Library_Utilities

Utility functions used by functions within `Vessel_Library`, e.g. to compute the isotropic undecimated wavelet transform used for segmentation. These do not have a fixed format for input and output arguments.

Vessel_Library_Dialogs

Additional custom dialog boxes and figures used by functions within `Vessel_Library` to obtain user input.

4.2 Designing new vessel processors

Each `.vessel_processor` file contains a MATLAB `struct` called `args`, which has a single compulsory field `args.processor_function` that contains the processing function name. Other fields can have any name and contain whatever extra data is required by the function.

The header for the processor function is

```
function [vessel_data, args, cancelled] = processor_function(
vessel_data, args, prompt)
```

where the first argument is a `Vessel_Data` object containing the image to be processed, the second is the `args` structure and the third is either `true` or `false` depending upon whether the user should be prompted for input. If this is `true`, then where possible the user should be requested for any values that should be stored in the `args` structure, which will then be saved in the processor after the processing is complete.

The first two output arguments are the same as the input arguments, but possibly modified by the function. If the user cancels the operation at some stage while prompting for settings then `cancelled` should be set to `true` so that the image is not displayed.

4.2.1 Duplicating existing processors

If a new processor should share the same function as an existing processor, simply duplicate the file, select it in the `Processors` menu, and open an image with the `Prompt for settings` option checked to change its arguments.

4.2.2 Writing new algorithms

The `struct` for a new processor can be created at the MATLAB command prompt, and stored using the `save_vessel_processor` command. Any `.m` file on the MATLAB search path may be used as the `processor_function`, provided it uses the same input and output arguments as described above and it sets all the necessary properties of the `Vessel_Data`. An example is given in `aria_algorithm_general.m` . Any such function put into the `Vessel_Algorithms` folder can be added to a processor with the `Processors` → `Create new processor` command.
